# A Monitoring System for the Segmentation and Grading of Broccoli Head Based on Deep Learning and Neural Networks

**DOI:** 10.3389/fpls.2020.00402

**Published:** 2020-04-15

**Authors:** Chengquan Zhou, Jun Hu, Zhifu Xu, Jibo Yue, Hongbao Ye, Guijun Yang

**Affiliations:** ^1^Institute of Agricultural Equipment, Zhejiang Academy of Agricultural Sciences, Hangzhou, China; ^2^International Institute for Earth System Science, Nanjing University, Nanjing, China; ^3^Key Laboratory of Quantitative Remote Sensing in Agriculture of Ministry of Agriculture P. R. China, Beijing Research Center for Information Technology in Agriculture, Beijing, China; ^4^National Engineering Research Center for Information Technology in Agriculture, Beijing, China

**Keywords:** growth monitoring, deep learning, improved ResNet, freshness grading, ground-based imaging system

## Abstract

Achieving the non-contact and non-destructive observation of broccoli head is the key step to realize the acquisition of high-throughput phenotyping information of broccoli. However, the rapid segmentation and grading of broccoli head remains difficult in many parts of the world due to low equipment development level. In this paper, we combined an advanced computer vision technique with a deep learning architecture to allow the acquisition of real-time and accurate information about broccoli head. By constructing a private image dataset with 100s of broccoli-head images (acquired using a self-developed imaging system) under controlled conditions, a deep convolutional neural network named “Improved ResNet” was trained to extract the broccoli pixels from the background. Then, a yield estimation model was built based on the number of extracted pixels and the corresponding pixel weight value. Additionally, the Particle Swarm Optimization Algorithm (PSOA) and the Otsu method were applied to grade the quality of each broccoli head according to our new standard. The trained model achieved an Accuracy of 0.896 on the test set for broccoli head segmentation, demonstrating the feasibility of this approach. When testing the model on a set of images with different light intensities or with some noise, the model still achieved satisfactory results. Overall, our approach of training a deep learning model using low-cost imaging devices represents a means to improve broccoli breeding and vegetable trade.

## Introduction

Broccoli (*Brassica oleracea* L. var. *italica*), which is belongs to the genus *Brassica* in the family Cruciferae, is considered as an important global vegetable crop. The present broccoli cultivation area in China is about 140,000 hectares. Broccoli is an important export vegetable in China, especially in Zhejiang Province (Kalisz et al., 2015). The broccoli head is an important agronomic component, which is not only used for yield estimation but also to assess plant quality and analyze plant resistance to biotic and abiotic stresses (Guo et al., 2017). A variety of techniques have been used for the quantitative measurement of broccoli head, including the destructive measurement of geometric parameters and dry weight, mass spectrometric analysis, and techniques using non-contact sensors. Destructive techniques are not suitable for the measurement of broccoli head under a controlled environment due to its low-throughput and unsustainability. Mass spectrometric analysis has been applied to measure the quality and freshness of vegetation, however, the wide application of this method is restricted by its high cost (Cho et al., 2018). The sustainable monitoring of broccoli head can be achieved through various technological innovations such as non-contact sensors. Non-contact sensors, which are principally based on digital RGB cameras, are suitable for use in agriculture due to their high resolution, low cost, and small size. The use of RGB cameras can provide a non-invasive and high-throughput approach to collect morphological information about broccoli head and analyze its health status (Dell’ Aquila, 2009). Changes in soil reflection and weather conditions, as well as the transition between growth stages, will all cause differences in the reflectivity, size, shape, and color of a broccoli canopy. The existing segmentation methods based on RGB images are of two main types: (1) those solely based on color information; and (2) those based on multi-features and a trained model (Hamuda et al., 2016). For example, for the first type, Ji et al. (2007) presented a real-time segmentation algorithm for plant images under natural outdoor conditions by using a threshold-based method. Their experimental results demonstrated that segmentation was generally of good quality in the case of bare soil background. Furthermore, Wang et al. (2013) established relationships between image feature parameters and several plant indexes by setting threshold values based on magnitude. The high correlation coefficients (over 0.9) which were achieved between the segmented canopy cover and the selected plant indexes indicate that this technique could be used to estimate the nitrogen status of vegetation. However, due to the complexity of field environments, color information will be seriously affected by the illumination intensity or shadows. In the second type of segmentation method, regions of interest are generated by extracting multi-features and training a classifier. Islam et al. (2017) used a support vector machine (SVM) classifier to realize the automated detection of potato diseases with an accuracy of 95%, which presented a path toward the automated diagnosis of plant diseases at a very large scale. Additionally, Yang et al. (2016) proposed a novel plant-inspired optimization algorithm which essentially mimics iterative root foraging behaviors, named the “hybrid artificial root foraging optimizer,” in order to determine root density. Cates et al. (2004) developed a novel leaf-segmentation tool by combining *a priori* information with local images showing the orientations of leave. The approach of Cates et al. (2004) achieved higher accuracy compared to three other state-of-the-art segmentation techniques. Moreover, Chantal et al. (2014) used an improved method based on a non-destructive and high-throughput machine learning method to accomplish non-contact analysis in order to measure root architecture. Previous methods for the segmentation of plant utilized handcrafted features such as shape, color, and texture to quantify the pixel character of plants. Extracting such features often requires some theoretical knowledge of botany and a computationally expensive preprocessing step in order to enhance differences between plants and background, i.e., an image binarization step (Wang and Xu, 2018). To allow simple and effective segmentation, most studies based on botanical theories use images captured in a controlled environment with a uniform background.

Furthermore, in recent years, convolutional neural networks (CNNs) have matured and have revolutionized computer vision. Currently, CNNs achieve superior results in the identification and segmentation of plants compared with state-of-the-art traditional methods. CNNs have been used to improve the performance of the approach for identifying and counting plant species, to quantitatively phenotype plants grown in controlled environments, and to provide detailed quantitative characterization of fruits and leaves. For example, Jeon et al. (2008) proposed an image segmentation method to detect individual weeds using color space transformation, threshold calculation, and the training of an artificial neural network (ANN). Additionally, Yamamoto et al. (2014) developed a method for the accurate detection of individual intact tomato fruits by using a conventional RGB digital camera in conjunction with machine learning approaches. Moreover, Xiong et al. (2017) used a rice panicle segmentation algorithm, called Panicle-SEG, to segment and calculate of rice panicle, and performed phenotyping of rice panicle to assist rice breeding. The overall accuracy of their model (more than 0.89) demonstrated the practical utility of their model for the estimation of field yield. Furthermore, Zhang and Xu (2018) introduced an unsupervised field image segmentation algorithm, called Unsupervised Learning Conditional Random Field (ULCRF), to accelerate the unsupervised segmentation of greenhouse plant images at the organ level. Kusumam et al. (2017) describe a 3D vision system for robotic harvesting of broccoli using low-cost RGB-D sensors. Ramirez (2006) develop a computer vision algorithm to locate the broccoli head within an image of an entire broccoli plant and has the ability to distinguish between mature and immature broccoli heads. However, despite the improvements which have been achieved in deep learning, the accurate segmentation and grading of broccoli requires a large amount of training data and depends on the quality of the images used. For field-based imaging and analysis systems, it is important to overcome the lack of training data and low processor capacity to achieve a high-throughput phenotype acquisition process (Lee et al., 2018).

Therefore, the aim of this study was to develop an automatic method to segment near-ground RGB images of broccoli field in order to extract broccoli head and construct a high-throughput grading standard. Some major advantages of this approach are that it requires only a few images and reduces the data volume and memory requirements for the image processing. This approach also allows the use of deep learning technology which is not specific to agriculture and plant phenotyping. Compared with three other approaches, the evaluation results showed better performance regarding the segmentation and grading accuracy.

## Materials and Methods

### Experimental Setup

All experiments were conducted at the Zhejiang Academy of Agricultural Sciences (ZAAS) Yangdu Scientific Research Innovation Base, Haining County, Zhejiang Province, China (latitude 30°27′ N, longitude 120°25′ E). Soil samples were collected from a depth of 0–20 cm with a pH range from 4.5 to 6.5 and organic matter content of more than 30 g kg^–1^. Other soil chemical properties of the experimental area are as follows: the available phosphorous content was 20 mg kg^–1^, the content of rapidly available potassium was 300 mg kg^–1^, and alkali-hydrolyzable nitrogen was 300.2 mg kg^–1^. The broccoli samples consisted of three varieties, named *Zheqing95*, *Tailv1*, and *Tailv2* which are the main broccoli planting varieties in Zhejiang Province, and each variety was planted in a separate plot. All plants were directly artificially seeded on 15 September, 2018. The three experimental plots consisted of raised beds with a length of 20 m and a width of 1 m with the seed lines separated by 0.3 m. The inter-row distance was 0.8 m and the inter-plant distance was 0.35 m.

### Remote Monitoring System and Image Acquisition

**FIGURE 1:**
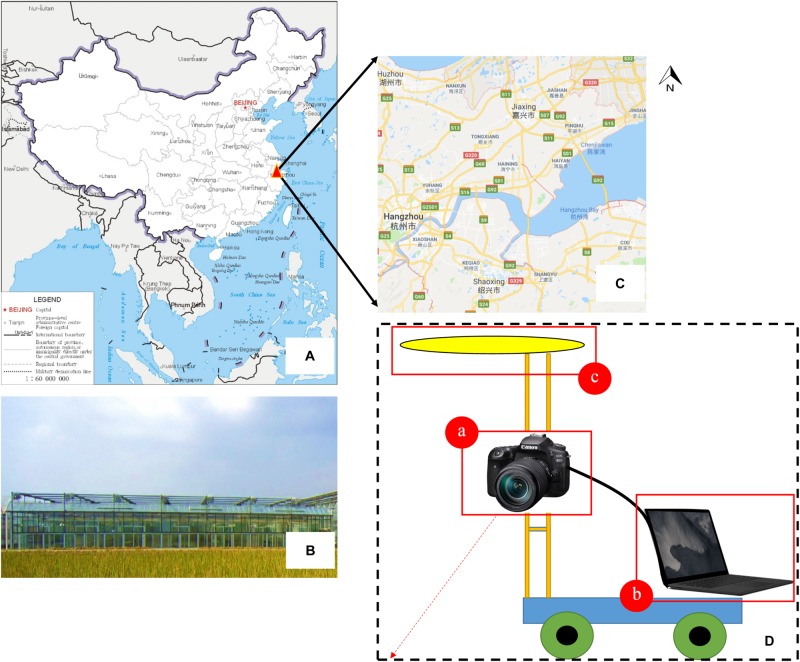
Part of the trial design. **(A–C)** Test site location and the appearance of the greenhouse; **(D)** field imaging system and integrated sensors**—(a)** Canon EOS 90D digital camera, **(b)** Surface Laptop 2 computer, **(c)** uniform LED surface light source.

The vision system was composed of an Canon EOS 90D camera (Canon, Inc., Tokyo, Japan) with a resolution of 32.5 megapixels (6960 × 4640 pixels) and a 22.3 mm × 14.8 mm CMOS sensor, a uniform LED surface light source, and a Surface Laptop 2 computer (Microsoft, Corp., Redmond, WA, United States) with an Intel Core i7-8650U processor and 8 GB Samsung DDR4 memory. All of these devices were mounted on a semi-automatic field self-propelled platform with dimensions of 1.5 m × 1.5 m × 1 m. The component elements of the surface light source were 120 white LEDs (3000 K) arranged in a circular pattern. The camera was controlled by an electronic shutter connected to the computer by a USB 3.0 interface. The computer installed with a visualization software named “pylon Camera Software Suite” (BASLER, Inc., Ahrensburg, Germany) was used to monitor the quality of the images in real time and guarantee the data quality. The camera was located 1.5 m from the ground level with the focal length of 18 mm and the exposure time of 1/500 s. The platform moved with a speed of 1 m/s, and the field of view was about 0.2 m^2^, which generated an image sequence with an average overlap of more than 70%. Two data acquisitions were conducted for each of the three plots. In total, 506 images containing grown broccoli plants were captured, and these were used to build our original image datasets. Among these images, 300 images were obtained during our first data acquisition, which was called “T1,” while the rest of the images were captured during the second acquisition, which was called “T2.” In this way, we can make our dataset contains plant images with different flower-ball shape, color distribution and hollow degree (Figure 1).

### Image Preprocessing

**FIGURE 2:**
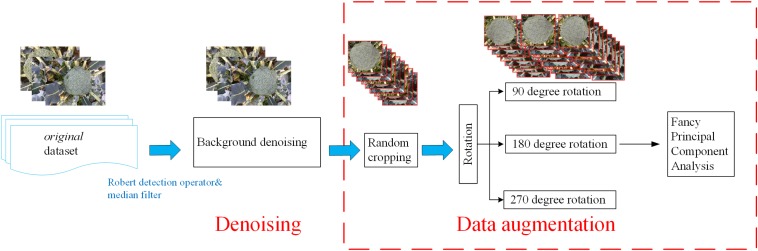
Flowchart of the image preprocessing.

The plant images captured by the self-developed monitoring system were pre-processed to denoise the background and enhance the images. This pre-processing was performed via several python-based scripts (Figure 2).

#### Background Denoising

Field images typically contain various sources of noise, which will affect the final training results. In order to remove high-frequency noise from the images, a Robert detection operator was applied to extract the edge of the broccoli image (Chaudhuri and Chanda, 1984), followed by a median filter with a size of 3^∗^3 pixels to remove the noise from the images (according to the size of broccoli head and flower bud displayed in the images) (Zheng et al., 2017).

#### Data Augmentation

A sufficiently large training dataset is essential to improve the final accuracy of deep-learning projects [19]. However, in our study of broccoli heads, it was not possible to capture enough images due to the limitations of the indoor environment. Therefore, in order to improve the quantity and quality of the training images, data enhancement methods were adopted to expand the dataset by 12 times (show in Table 1).

**TABLE 1:** Dataset configuration.

**Variety**	**Original**	**Augmentation**	**#Train**	**#Val/Test**
*Zheqing95*	165	2000	1500	250/250
*Tailv1*	171	2000	1500	250/250
*Tailv2*	170	2000	1500	250/250

##### Random cropping

First, the original images were resized to 1440 × 1080 pixels due to the memory limitation of the GPU. Then, three sub-images with a size of 480 × 360 pixels were randomly cropped from each image. Thus, the number of training images was tripled.

##### Rotation

In order to further increase the number of training images, the cropped images were rotated by 90, 180, and 270 degrees, respectively, thus increasing the size of the dataset by a further four times.

##### Fancy principal component analysis

The last step in the data enhancement procedure involved applying a fancy principal component analysis (FPCA) algorithm to change the intensity of the RGB channels in order to enhance the contrast between the broccoli heads and the background (Morais et al., 2019). The feature vectors were generated by the FPCA algorithm, and then a weight factor was added to the corresponding channel according to the extracted feature vector. The use of FPCA can transform the illumination intensity and color of the image without affecting the object to be recognized.

### Data Analysis

**FIGURE 3:**
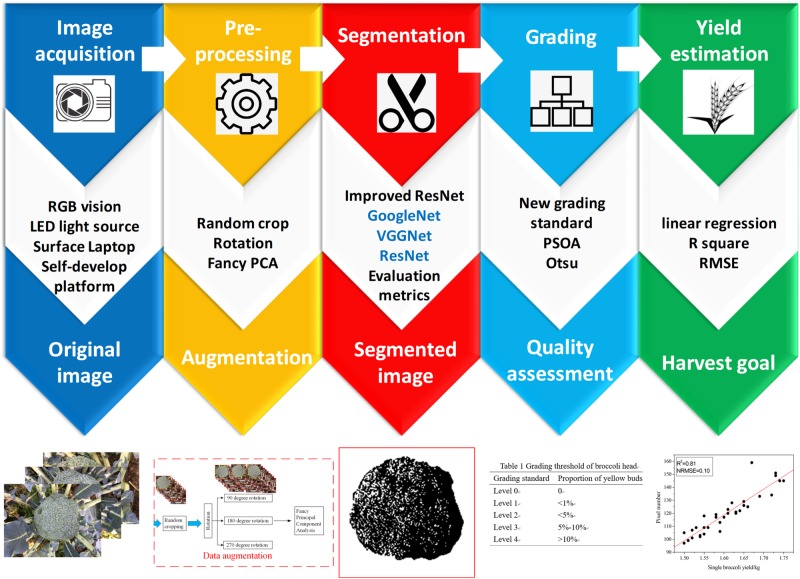
The flow of the proposed method.

The purpose of this study was to develop a general system for the automatic detection and grading of broccoli head. This system requires the input of orthophoto images of the field canopy and outputs the segmentation results generated by a novel “Improved ResNet” model and grading results determined by a pixel clustering method. The overall flow of the method is shown in Figure 3. Our approach comprises three steps: (1) detect the positions of the broccoli heads and calculate the orthophoto projection area; (2) establish a yield estimation model based on the correlation between flower-ball area and weighing results; and (3) conduct flower-ball grading based on a pixel clustering algorithm.

#### Architecture of the Training Models

The growing broccoli heads were detected using a CNN named “Improved ResNet.” In order to verify the accuracy of the model, three classical deep learning architectures were used for comparative experiments, namely GoogleNet, VGG16, and ResNet 50. The open-source codes of these approaches were implemented under the TensorFlow framework.

##### GoogleNet

GoogleNet reduces the number of filters and training parameters which are required compared with the traditional Inception Structure (Tang et al., 2016). Additionally, it maximizes the depth and width of the network and divides the multidimensional convolution layer in the Inception Module into several smaller one-dimensional convolution layers by decomposition factor, which not only reduces the number of parameters in the model, but also effectively avoids over-fitting.

##### VGGNet

The VGG network constructs a CNN with a depth of 16/19 layers by repeatedly stacking small convolution cores with a size of 3^∗^3 and maximum pooling layers with a size of 2^∗^2 (Mehdipour Ghazi et al., 2017). In this study, the VGG16 network model was adopted; the number 16 denotes the number of layers using convolution layer besides pooling layer. VGG16 uses convolution blocks consisting of 2–3 convolution layers so that the network has more receptive fields and fewer network parameters, and also uses a Rectified Linear Unit (ReLU) activation function to perform numerous linear transformations to achieve greater learning ability.

##### ResNet

**FIGURE 4:**
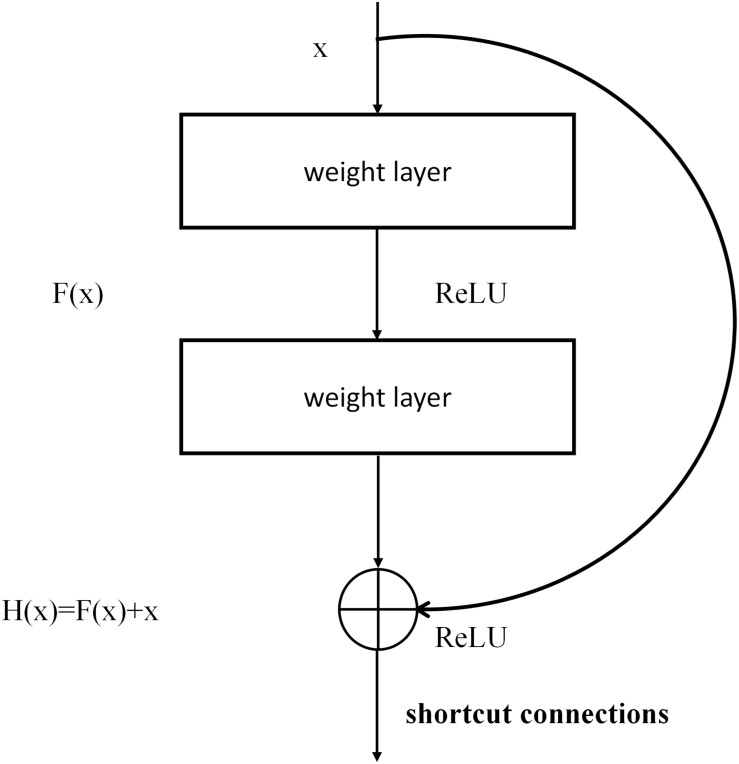
The structure of residual module in ResNet.

ResNet is a complete network formed by the repeated accumulation of residual learning modules (He et al., 2016). The introduction of residual modules solves the problem of gradient dispersion and enhances the feature-learning ability and recognition performance. The structure of the residual modules is shown in Figure 4. Set *x* as the input and *F* (*x*, W_1_, W_2_) as the output after the convolution of W_1_ and W_2_ (the weighting parameters to be learned). The activation function is set as ReLU, so the final output of the residual module unit *y* can be expressed as follows:

(1) $$y=F\,\left(x,W_{1},W_{2}\right)+W_{s}\,x$$

where W_1_ and W_2_ represent the weighting parameters to be learned, and W_s_ represents a square matrix that transforms *x* from the input residual module dimension to the output dimension.

##### Improved ResNet

In supervised learning mode, a large amount of data is needed to train the residual network model. However, at present, few broccoli images are available with labels, which cannot meet the needs of training deep network models. Therefore, in order to improve the accuracy and generalization ability of the ResNet-50 model, a feature- based transfer learning was adopted which combines transfer learning and deep learning. First, ImageNet was used to pre-train the ResNet-50 network to allow it to extract image features, and the trained network parameters were used as network models. Then, broccoli-head images were precisely segmented by adjusting the parameters of the ResNet-50 network. A three-layer adaptive network was used to replace the full connection layers and the classification layers of the ResNet-50 model, and the LReLUSoftplus was adopted as the activation function of the architecture. The formulas for ReLU and LReLUSoftplus are given as follows.

**FIGURE 5:**
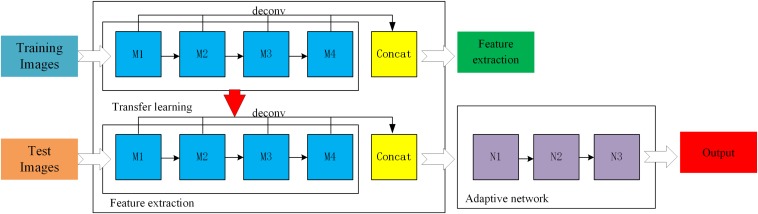
A general scheme of the Improved ResNet.

(2) f⁢(x)=max⁡(0,x)

(3) $$f\,\left(x\right)=\left\{ \begin{array}{c} \ln\left(e^{x}+1\right)-\ln2,x\geq 0\\ \text{ax},x<0 \end{array}\right.$$

where x indicates the input value, and *a* is set as 0.01. A general scheme of the proposed method is shown in Figure 5. M_1_, M_2_, M_3_, and M_4_ are the four residual blocks in the ResNet-50 model, while N_1_, N_2_, and N_3_ are the three components of the adaptive network. In order to enrich the extracted features, all residual blocks were deconvoluted to get the corresponding features before the residual block convolution, then the deconvolution features were fused by using weighted fusion method.

The hyper-parameters for all experiments were as follows: the loss function was set to dice loss due to this function’s good performance in dichotomous problems; the base learning rate was 0.001 in the first 3000 iterations and was changed to 0.0005 in the subsequent 2000 iterations; the value of momentum and dropout were 0.9 and 0.8, respectively; and the number of epochs was 200 and the batch size was 64. We set the ratio of training images, validation images and test images to 6:1:1 in order to ensure the credibility of the training results.

#### Yield Estimation

After the canopy area of broccoli-head had been measured using the deep learning approach, a yield estimation model was developed by calculating the relationship between the weight of head balls (mean fresh matter of the broccoli heads within 2 h after harvest) and the segmentation area using regression analysis. Here, we treat the mean fresh weights of the broccoli heads and the mean value of the projected area of the corresponding variety as the independent and dependent variables of the formulas, respectively.

#### Grading System

An artificial grading standard for broccoli based on the sensory yellowness of the flower-ball was adopted from Tu et al. (2007). The details of the grading standard are shown as follows:

Level 0: unable to detect yellow flower buds;
Level 1: 1–3 yellow flower buds detected;
Level 3: detect 5% yellow area;
Level 5: 50% yellow area detected;
Level 7: yellowness degree between 50 and 75%;
Level 9: all the detection area displayed yellow color;

The yellowness degree of broccoli head can be calculated by Eq. 4:

(4) $$Y=\sum M_{1}*N_{1}/M_{2}*N_{2}$$

where *Y* represents the degree of sensory yellowness, *M*_1_ represents the level of each broccoli under our grading system, *N*_1_ is number of the broccoli in the corresponding level, *M*_2_ is the highest level, and *N*_2_ is the total number of observed broccoli. Since it was only necessary to distinguish two types of color in our research (green and yellow) and these two parameters were completely different, it was convenient to separate them by using a threshold segmentation technology. In this paper, an optimized Otsu method was used to transform the grayscale images of the head ball into two parts and calculate the number of black and white pixels respectively to determine the proportion of yellow area. In our practical experience, it was not necessary to achieve such precise grading since broccoli heads with more than 10% yellowness area has no commercial value to any consumers. We merged Level 4 (10% yellow area detected) to Level 9 and then divided all the broccolis into five levels according to the new standard (Table 2).

**TABLE 2:** Grading threshold for broccoli head.

**Grading standard**	**Proportion of yellow buds**
Level 0	0
Level 1	<1%
Level 2	<5%
Level 3	5–10%
Level 4	>10%

#### Data Annotation

For the supervised learning algorithm, the quality of ground truth determines the accuracy of the final results. In the field of image segmentation, in addition to some cases where open datasets are available, many application scenarios require specialized datasets for migration learning or end-to-end training. The methods for constructing ground truth datasets can be separated into three categories: manual labeling, automatic labeling, and outsourcing labeling. Among them, automatic labeling usually requires a second review to avoid program errors, and outsourcing labeling introduces the risk of data leakage and loss. Meanwhile, manual labeling is usually time consuming, although the results are relatively reliable. In our study, the manual works were conducted by four people using the Labelme tool to draw curved lines to precisely segment the broccoli heads. The code of this tool^1^ is open source so that it can be used by anyone to build a labeled training dataset.

### Evaluation Index

The performance of our segmentation model was evaluated using five different metrics: (1) Accuracy; (2) Precision; (3) Intersection over Union (IOU); and (4) Recall and (5) F-Measure. “Accuracy” is the proportion of correctly extracted flower ball pixels to the total number of pixels. The higher the value (approach to 1), the more accurate the segmentation is. IOU is applied to describe the degree of ratio of intersection and union of real and predicted values. Precision and Recall can be used to reveal accuracy and the completeness of the segmented region. These two indexes interact with each other, and the F-measure was used to balance them. The computational formulas of these five evaluation indexes are shown in Eqs 5–9:

(5) $$A\,c\,c\,u\,r\,a\,c\,y=\frac{T\,P+T\,N}{T\,P+T\,N+F\,P+F\,N}$$

(6) $$I\,O\,U=\frac{P\,r\,e\,d\,i\,c\,t\,e\,d\cap G\,r\,o\,u\,n\,d\,T\,r\,u\,t\,h}{P\,r\,e\,d\,i\,c\,t\,e\,d\cup G\,r\,o\,u\,n\,d\,T\,r\,u\,t\,h}$$

(7) $$P\,r\,e\,c\,i\,s\,i\,o\,n=\frac{T\,P}{T\,P+F\,P}$$

(8) $$R\,e\,c\,a\,l\,l=\frac{T\,P}{T\,P+F\,N}$$

(9) $$F-M\,e\,a\,s\,u\,r\,e=\frac{2*P\,r\,e\,c\,i\,s\,i\,o\,n*R\,e\,c\,a\,l\,l}{P\,r\,e\,c\,i\,s\,i\,o\,n+R\,e\,c\,a\,l\,l}$$

where, TP, TN, FP, and FN in Eqs 5–7 represent the numbers of true positives, true negatives, false positives, and false negatives, respectively. Among them, the true positives represent the extracted pixels and the corresponding ground truth which both belong to the flower ball region. True negatives are when extracted pixels and the corresponding ground truth are both background pixels. False positives are when the pixels are classified as flower ball pixels but the ground truth results display them as background pixels. False negatives represent the background pixels that are not correctly discriminated. The “*Predicted*” in Eq. 6 represents the prediction results achieved by these segmentation algorithms.

## Results and Discussion

The experiment was conducted on images of growing broccoli which were captured by a camera mounted on a self-developed near-ground imaging system equipped with a series of auxiliary imaging devices. Using our post-processing system (Microsoft Windows 10 Professional operating environment with a 12-core Intel Core i7-8700K CPU, 16 GB of memory, and an NVIDA GTX 1080Ti video card), the segmentation process for one test image with a resolution of 1440^∗^1080 only takes 1.5 s. Moreover, by applying compute unified device architecture (CUDA) parallel acceleration model, the average processing time of a single image could be increased to 0.7 s. The performance of the developed deep learning method was evaluated using the evaluation metrics mentioned in Section “Evaluation Index,” by comparison to manual ground truth results. All of the models presented in this paper are based on Python.

### Broccoli Head Segmentation

**FIGURE 6:**
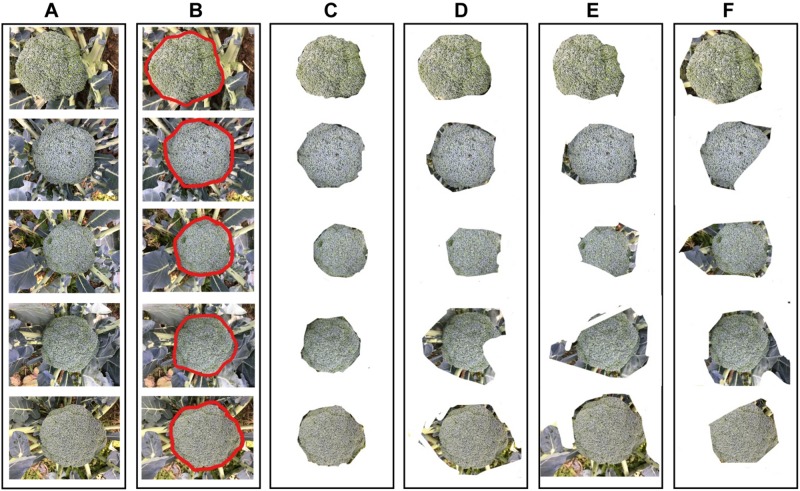
The results of broccoli head segmentation using different approaches. **(A)** Original images, **(B)** annotation results **(C)** segmented by Improved ResNet, **(D)** segmented by GoogleNet, **(E)** segmented by VggNet, and **(F)** segmented by ResNet.

**FIGURE 7:**
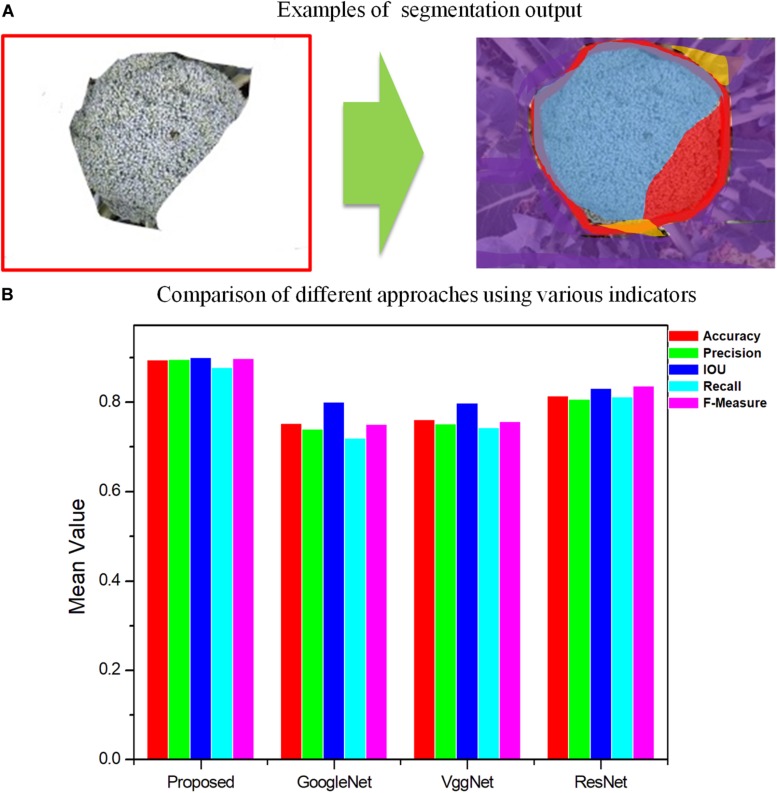
Comparison of different approaches by segmentation quality for GoogleNet, VggNet, ResNet, and the proposed method, Improved ResNet. **(A)** Examples of segmentation output. Blue region-TP, Red region-FN, Orange region-FP, Purple region-TN**. (B)** Comparison of different approaches using various indicators.

The broccoli segmentation method was tested using the whole test set. Some of the results are presented in Figure 6. In Figure 6, three representative testing images were chosen to show the segmentation results obtained using different approaches. For each broccoli, the original image is shown in Line A and the segmentation results for each compared model are shown in Lines C–F. To demonstrate the accuracy and robustness of deep learning for plant monitoring, the evaluation metrics of Accuracy, Precision, IOU, Recall, and F-Measure were analyzed, as shown in Figure 7. In this figure, the color columns represent the mean value of the evaluation metrics. The color differences between these columns represent the various indicators. All the statistical analysis was performed using the SPSS 19.0 software (IBM, Inc., Armonk, NY, United States) (Figures 6, 7).

As shown in Figures 6, 7, for Improved ResNet, the average Accuracy and Precision for the test images are about 0.896 and 0.897, which are higher than the values obtained using the three other contrast algorithms. Additionally, the proposed method can achieve better consistency with labeling results. Furthermore, the high IOU of 0.901 of the proposed method shows a high overlapping rate between the candidate region and the labeling area. The IOU values of the other CNN models were as follows: GoogleNet, 0.801; VggNet, 0.799; and traditional ResNet, 0.832. A mean value of Recall of 0.879 can be achieved by using the Improved ResNet, compared with 0.721, 0.744, and 0.813 for GoogleNet, VggNet, and ResNet, respectively. Moreover, as illustrated in the figure, a higher F-measure can be obtained using Improved ResNet (F-measure is a comprehensive indicator that accounts for Recall). Compared with the other approaches—which achieved relatively lower F-measure values, with values of 0.751, 0.758, and 0.838 achieved using GoogleNet, VggNet, and ResNet, respectively—the Improved ResNet had a higher mean F-measure (0.899) with a lower standard deviation. This shows that the proposed algorithm was able to accurately distinguish broccoli head regions from the background region and guarantee the integrity of flower-ball structural information.

### Yield Estimation Results

**FIGURE 8:**
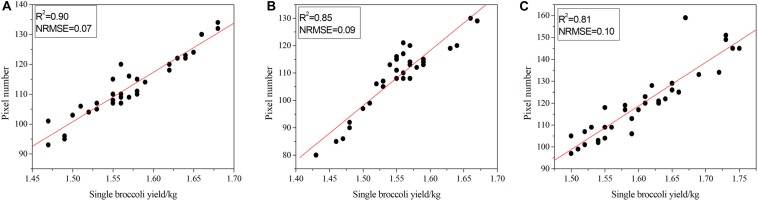
Correlation between pixel number and the quality of broccoli. **(A)** Correlation results for *Zheqing95*, **(B)** correlation results for *Tailv1* and **(C)** correlation results for *Tailv2.*

All of the broccolis were harvested on 6 January 2019, and the quality of each plant was recorded by skilled workers. The test set contained a total of 100 broccolis, which were arranged and numbered in order of weight and then split into an odd group and an even group, which were used for modeling and verification, respectively. In this study, linear regression was used to realize non-destructive production estimation. The number of pixels occupied by the head of broccoli as obtained by the segmentation algorithm was used as the independent variable, and the quality of a single broccoli was treated as the dependent variable. To quantitatively assess the performance of the linear regression algorithm, various popular elevation metrics were computed, namely the determination coefficient (R^2^) and the normal root-mean-squared error (NRMSE). These indexes have been widely used to estimate the predicative power of regression models. A larger R^2^ indicates a better fitting of the model, while a smaller NRMSE indicates a better estimation. We defined R^2^ and NRMSE as follows:

(10) $$R^{2}=\frac{1-S\,S\,E}{S\,S\,T}$$

(11) $$N\,R\,M\,S\,E=\frac{\sqrt{\frac{1}{N}\,\sum_{i=1}^{N}{\left[Y\,\left(i\right)-Y^{\prime }\,\left(i\right)\right]}^{2}}}{\bar{Y}}$$

relating the obtained number of pixels with the quality of each broccoli. In Eq. 10, *SSE* represents the *Sum of Squares for Error* and *SST* represents the *error sum of squares*. In Eq. 11, *N* and *Y*′(*i*) correspond to the number of samples and the actual value of sample *i*, respectively; *Y*′(*i*) represents the estimated value of sample *i*; and $\bar{Y}$ is the average actual value of the sample. For regression analysis, the number of pixels identified for the three species of broccoli was selected as the independent variable, and the corresponding individual weight was used as the dependent variable. The regression model conducted with our test set is shown in Figure 8.

As illustrated in Figure 8A, for *Zheqing95*, the number of pixels was strongly correlated with manual measurements of plant quality (adjusted *R*^2^ = 0.90), which is consistent with the result for NRMSE (adjusted NRMSE = 0.07). As shown in Figure 8C, the lowest R^2^ occurs with the largest NRMSE of *Tailv2*. These differences seem to be strongly species-specific, even in the same growth stage. Compared with the accurate quality results, the image-based yield estimation had two additive error sources: (1) the head region was covered by leaves or insects; and (2) misclassification of head pixels and background pixels. In the first situation, the estimated production may be higher than the actual production due to the loss of pixel statistics. This is a technical limitation, and the RGB camera cannot remove the occluded objects from a certain view. A possible solution is to use opening and closing operations to remove these small holes in the head; however, this may not suitable for broccolis with large flower buds. An alternative solution is to recognize the leaves, etc., independently, which will greatly increase the complexity of the model. In the second situation, the number of pixels that presented a certain plant is larger than the real value. This issue can be solved by providing more training samples and fine-tuning the hyper-parameters to improve the accuracy of the pixel classification process.

### Grading Results Based on Image Analysis

After applying grayscale transformation and the Otsu algorithm, the fine classification of the broccoli-head pixels was carried out. In this study, the optimal threshold for each image was determined by using a traversal algorithm within a small range. The Particle Swarm Optimization Algorithm (PSOA) was used to determine the fitness function and fitness parameters (Wang et al., 2017). Based on the image analysis results and the new grading standard, we provide reliable grading results for broccoli quality (Table 3).

**TABLE 3:** Grading results for broccoli quality obtained by using the Particle Swarm Optimization Algorithm (PSOA) and the Otsu algorithm.

	**Level 0**	**Level 1**	**Level 2**	**Level 3**	**Level 4**	**Accuracy**
*Zheqing95*	31.2%	29.7%	12.5%	13.4%	13.2%	0.879
*Tailv1*	38.9%	37.7%	11.6%	7.8%	4.0%	0.853
*Tailv2*	25.1%	22.4%	25.3%	10.4%	16.8%	0.841

Then, we tested the accuracy of our method by comparing our grading results with manual annotation results. It was found that, using the grayscale transformation and improved Otsu algorithm, more than 80% of broccoli heads were graded correctly; specifically, for *Zheqing95*, *Tailv1*, and *Tailv2*, the prediction Accuracy was 0.879, 0.853, and 0.841, respectively compared with manual annotation results. Therefore, the performance of the proposed method is appropriate for practical use. Further research should focus on the introduction of roundness information and bud number to build a regression model (Table 4).

**TABLE 4:** Comparison of the analysis accuracy of the quality of broccoli head based on Particle Swarm Optimization Algorithm (PSOA) and the Otsu algorithm in different varieties.

	***Zheqing95***	***Tailv1***	***Tailv2***
Accuracy	0.879	0.853	0.841
IOU	0.855	0.844	0.843
Precision	0.825	0.894	0.899
Recall	0.838	0.841	0.873
F-measure	0.851	0.865	0.895

Further, an experimental comparison result was provided to show how much segmentation quality affects the grading results. With other approaches displayed relatively lower values, the Improved ResNet had higher mean value of evaluation indices which indicate that the better segmentation quality could improve the accuracy of grading results (Table 5).

**TABLE 5:** Comparison of the analysis accuracy of the quality of broccoli head using different segmentation models.

	**Improved ResNet**	**GoogleNet**	**VggNet**	**ResNet**
Accuracy	0.867	0.814	0.827	0.835
IOU	0.848	0.801	0.789	0.811
Precision	0.877	0.798	0.767	0.825
Recall	0.858	0.799	0.772	0.813
F-measure	0.867	0.768	0.797	0.809

### Robustness and Efficiency Analysis

**FIGURE 9:**
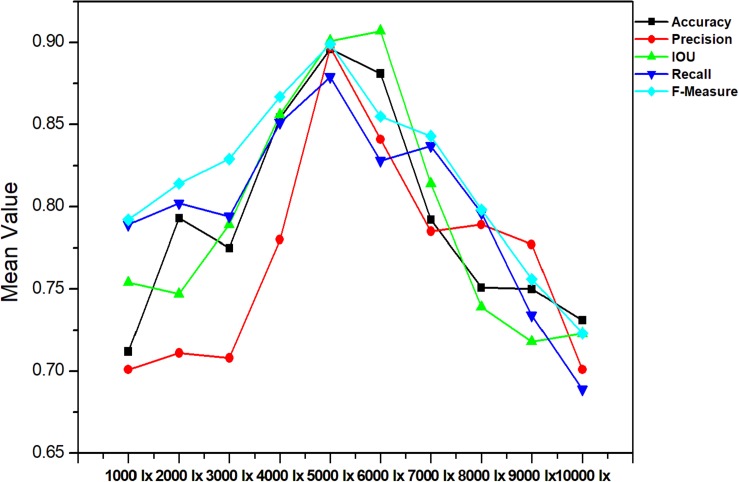
Performance of different segmentation methods under different light condition.

**FIGURE 10:**
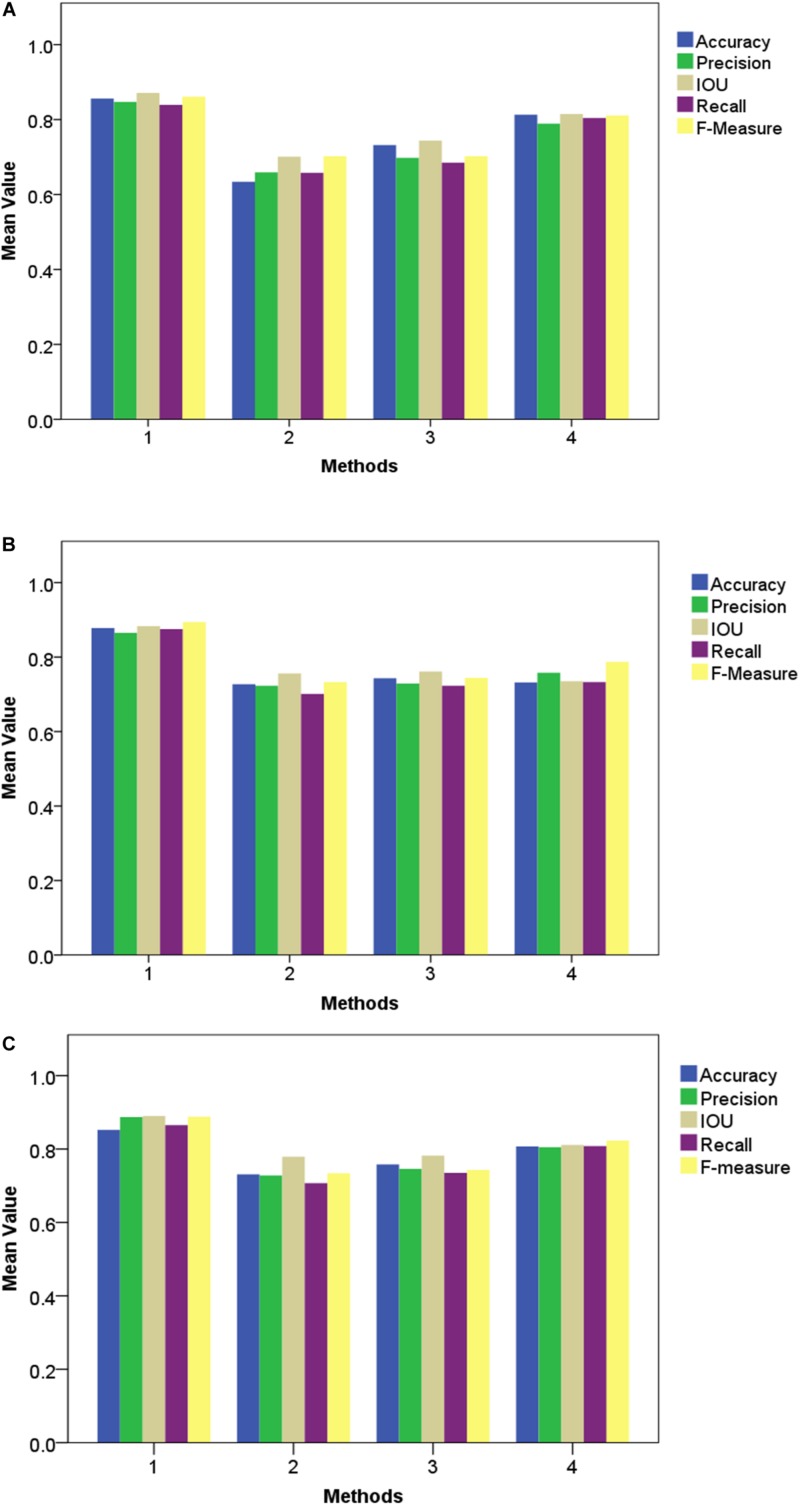
Performance of different segmentation methods under various types of noise interference. **(A)** Gaussian noise, **(B)** Salt and Pepper noise and **(C)** Rayleigh noise.

The accurate analysis of plant images acquired outdoors is a challenging task for researchers. Compared to pot experiments in the laboratory, segmenting field-grown vegetation is more complex due to varying light intensity, high specular reflectance, and ambient noise; each of these lead to the reduction of segmentation accuracy. Therefore, in order to be appropriate for the collection of plant phenotyping information, the segmentation method should be sufficiently robust to handle these unfavorable conditions. Figures 9, 10 present metrics indicating the quality of segmentation (Accuracy, Precision, IOU, Recall, and F-Measure) for the proposed method under different light condition (from 1000 to 10,000 lx) and under various types of noise interference (Gaussian noise, Salt and Pepper noise, Rayleigh noise). In comparison, GoogleNet, VggNet, and ResNet presented a similar analysis throughout our experiment.

As presented in these two experiments, although the three traditional architectures (GoogleNet, VggNet, and ResNet) can achieve satisfactory results under moderate brightness conditions, they performed poorly overall, particularly in circumstances with excessive or insufficient incident light. Additionally, the segmentation accuracy of GoogleNet and VggNet are greatly affected by Gaussian noise, while the traditional ResNet achieved much lower Recall and F-Measure when Salt and Pepper noise was introduced. The Improved ResNet achieved the highest mean Accuracy and the highest Precision, IOU, Recall, and F-Measure of the four compared methods. It must be noted that, we conducted all of our experiments in a confined environment (greenhouse etc.) because of the limitation of stability and power supply of our image acquisition platform. In other words, it is not a comprehensive robustness test for use under outdoor conditions. After the vehicle is further improved, we will apply it to outdoor conditions.

Moreover, the proposed algorithm achieved an average running time of 0.18 s for segmenting a single image, and thus represents a high-throughput processing method to measure the size of broccoli heads to inform decision-making in large-scale breeding. In our experiments, the 60 m^2^ field contained 1000s of broccoli plants, and the proposed method was able to calculate the area of the flower ball and grade all the plants within 30 s. Considering the target number of broccoli plants and image size, the Improved ResNet can adequately estimate biomass or yield for online measurement. The running time for each image ranges from 0.13 to 0.20 s. Additionally, we calculated the average time required for each step in the Improved ResNet procedure, and found that the most time-consuming step was segmentation. In our future work, we will attempt to improve hardware (including the I/O speed of the CPU and memory) in order to reduce the segmentation time.

### Limitations and Future Work

The imaging and processing procedure presented in this paper resulted in broccoli-head images with a high resolution, and allows the dynamic monitoring of the growth of individual broccoli plants at different growth stages. It must be noted that, the processing pipeline contains many steps which rely on manual settings and tuning which questions the wider applicability of the presented system. In the future, methods could be developed to improve the segmentation process in an attempt to eliminate the need for image labeling, such as semi-supervised learning (SSL) technology (Ma et al., 2015). In cases when there is only a small number of labeled samples, unlabeled samples can be labeled based on the similarity between unlabeled samples and labeled samples and the potential distribution of unlabeled samples and other strategies which will reduce the workload of annotation. Additionally, higher-resolution cameras would improve the overall process, albeit at the cost of increased processing time. Moreover, it is conceivable that an industrial robot could be used to perform both broccoli monitoring and automated harvesting based on the results of image processing, however this may become excessively cost prohibitive.

## Conclusion

In this study, we establish a robust image processing method for segmenting and grading broccoli head in field conditions based on a deep learning architecture and color information. Compared with three other approaches—namely GoogleNet, VggNet, and ResNet—the proposed Improved ResNet algorithm has better segmentation performance and grading accuracy. Moreover, our model was tested under different light intensities and noise categories to confirm its applicability. Realizing accurate segmentation is merely the first step, being a prerequisite for extracting image-based traits. Based on our experimental results, many other traits, such as Above Ground Biomass (AGB), yield, and biologic rhythm, could be obtained using the Improved ResNet algorithm. Therefore, this algorithm represents a powerful tool for the large-scale phenotyping analysis of broccoli in a non-invasive and automatized way, and could potentially facilitate breeding research in future.

## Data Availability Statement

The datasets generated for this study are available on request to the corresponding author.

## Author Contributions

CZ developed the detection and measurement algorithm, implemented the existing methods for comparison purposes, conducted the comparisons, obtained the results, and drafted the manuscript. GY developed the stomata detection algorithm. HY supervised the project, identified the main goals of the project, and contributed to writing the manuscript. JH and JY prepared the images for the research. ZX provided expert supervision for marking the ground-truth. All authors read and approved the final manuscript.

## Conflict of Interest

The authors declare that the research was conducted in the absence of any commercial or financial relationships that could be construed as a potential conflict of interest.

## Footnotes

1 https://github.com/wkentaro/labelme
